# Colonoscopy surveillance in Lynch syndrome is burdensome and frequently delayed

**DOI:** 10.1007/s10689-023-00333-4

**Published:** 2023-05-12

**Authors:** Elsa L. S. A. van Liere, Imke L. Jacobs, Evelien Dekker, Maarten A. J. M. Jacobs, Nanne K. H. de Boer, Dewkoemar Ramsoekh

**Affiliations:** 1 Department of Gastroenterology and Hepatology, Amsterdam University Medical Center location Vrije Universiteit Amsterdam, De Boelelaan 1117, Amsterdam, The Netherlands; 2 Department of Gastroenterology and Hepatology, Amsterdam University Medical Center location University of Amsterdam, Meibergdreef 9, Amsterdam, The Netherlands; 3 Amsterdam Gastroenterology Endocrinology Metabolism (AGEM) Research Institute, Amsterdam, The Netherlands; 4 School of Medicine, Vrije Universiteit, Amsterdam, The Netherlands

**Keywords:** Lynch syndrome, Colonoscopy, Surveillance, Compliance, Burden, Biomarker

## Abstract

Individuals with Lynch syndrome have an increased colorectal cancer risk, hence, biennial colonoscopy surveillance is recommended. We aimed to investigate patients’ perception and preferences regarding surveillance, and to further explore compliance behaviour. Individuals with Lynch syndrome received a validated survey evaluating experiences of their three most recent colonoscopies. Individuals were non-compliant to surveillance if the interval between colonoscopies differed ≥ 6 months from the recommended interval. In total, 197 of 291 (68%) invited individuals returned the survey. They mostly underwent colonoscopy biennially (99%), under mild sedation (79%) and with bowel preparation performed by Moviprep® (99%). Surveillance was perceived as impacting quality of life in 21%, and as moderately to extremely burdensome in 57%, particularly in those below age 40. To lower the burden, patients prioritised improvements in volume and taste of bowel preparation, laxation-related bowel movements, waiting times, and a more personal and respectful approach of endoscopic staff. Additionally, many individuals (60%) would favour less-invasive surveillance modalities such as biomarkers. In total, 28% of individuals had delayed colonoscopy surveillance, predominantly for patient-related reasons. An additional 10% considered quitting/postponing surveillance. Upon multivariable analysis, patient-related delay was associated with low and medium education, history of ≤ 4 colonoscopies and having no hospital recall-system. Colonoscopy surveillance in Lynch syndrome is often experienced as burdensome, and frequently delayed. We identified determinants of surveillance behaviour in this population, and present potential interventions to reduce the burden and non-compliance rates.

**Supplementary Information:**

The online version contains supplementary material available at 10.1007/s10689-023-00333-4.

## Introduction

Individuals with Lynch syndrome, the most common hereditary colorectal cancer (CRC) syndrome, have a high lifetime risk for CRC, which varies between the different causal germline mutations from 15 to 70% [1]. Early detection of CRC, but also detection and removal of premalignant adenomas, reduces incidence, morbidity and mortality of CRC in Lynch [2]. Therefore, these individuals are advised to undergo biennial colonoscopy, starting from age 25 or 35 depending on mutated gene variant [1, 3–5]. Since the implementation of this surveillance program, the prognosis of individuals with Lynch syndrome has improved substantially [6, 7].

However, the effectiveness of a surveillance program for high-risk individuals is dependent on both the accuracy of the surveillance method as well as the adherence. Previous research has demonstrated that adherence to timely colonoscopy surveillance in individuals with Lynch syndrome is suboptimal [8–11]. As these studies are slightly outdated and do not reflect current, intensified surveillance recommendations, non-adherence rates might be even greater in clinical practice. Suboptimal compliance to colonoscopy surveillance can partly be explained by insufficient interval recommendations and suboptimal logistics, and by surveillance being performed in a non-specialty clinic and/or a clinic without coordination by a centralised (colorectal) cancer registry [8–10]. However, of greater influence are probably patients’ perceived barriers to invasive and regular colonoscopy surveillance, such as discomfort, embarrassment and surveillance being time-consuming [9, 10, 12, 13].

Multiple studies in average-risk individuals showed that colonoscopy and the required bowel preparation clearly come with a burden [14–16]. This burden might be even greater among individuals with Lynch syndrome, given that this high-risk population faces regular and lifelong colonoscopies and that surveillance for cancer on itself has a negative impact on psychological well-being and health related quality of life [17–20]. However, in this population no comprehensive study has yet investigated the perception of colonoscopy surveillance or factors that prompt or deter individuals to adhere to surveillance. Such detailed information is important to design targeted interventions to improve satisfaction with colonoscopy surveillance and lower its burden and non-compliance rates.

Therefore, the aim of the current study was to investigate the perception and preferences regarding different aspects of colonoscopy surveillance in a large cohort of individuals with Lynch syndrome. Secondly, we aimed to further explore surveillance behaviour in this population, including up-to-date non-compliance rates and predictors of (non-)compliance.

## Materials and methods

### Study design

In this cross-sectional study, we invited all individuals under colonoscopy surveillance for Lynch syndrome in an academic hospital in the Netherlands (Amsterdam UMC) with two different locations (location VUmc and location AMC). At the time of the study, location VUmc had an automated recall system for individuals with Lynch syndrome; individuals automatically received a telephone appointment with their physician to discuss and schedule colonoscopy when surveillance was almost due. Location AMC on the other hand did not have such a recall system and individuals had to contact the hospital themselves for a telephone appointment when they believed colonoscopy surveillance was almost due. Colonoscopies were performed on dedicated programmes by predominantly experienced residents, who were under direct supervision of twelve different experienced gastroenterologists (> 2000 colonoscopies). Some endoscopists worked at both location VUmc and location AMC, whereas others only worked at one of these locations. Both locations performed colonoscopies according to the national guidelines for Lynch syndrome, and used the same endoscopic equipment and colonoscopy protocol.

Only individuals over 18 years old with a proven germline mutation in one of the mismatch repair genes were included. To ensure a relatively similar surveillance method among study participants, individuals were excluded in case of surveillance by sigmoidoscopy or in case one of three most recent colonoscopies was performed in another centre. Eligible individuals received a letter by postal mail announcing the upcoming survey invitation, which would be send via e-mail using the platform Castor. Individuals who did not complete the survey within a week received a reminder per e-mail via Castor. The remaining non-responders were reminded by phone (if they had previously given consent to be contacted for endoscopy-related research), or otherwise received a second reminder per e-mail.

This study was approved by the Research Ethical Committee of Amsterdam UMC (2021.0516). Digital informed consent via Castor was obtained from all participants.

### Survey development

The survey for our study is based on validated surveys on the burden of colonoscopy or (CRC) screening programs that were used in previous studies [15, 16, 21–23]. Given that these validated surveys were now used in a slightly different study population and that some specific questions for the current population were added, in-depth interviews were conducted with four members of the Lynch patients’ organisation—in line with the COSMIN criteria [24]—to check the entire survey for relevance, comprehensiveness and comprehensibility. During these interviews, participants were asked to verbally express their thoughts while completing the survey (think aloud method [25]). After having processed the interviews, the optimised survey was sent to the four interview participants for a final check.

Our survey assessed different topics. First, the level of pain, embarrassment and burden associated with several aspects of colonoscopy (using a validated survey [15, 16, 21, 22] and five-point Likert scale). Individuals were asked to base answers on their three most recent colonoscopies or, if three were not yet performed, on all colonoscopies they had undergone. Second, the effect of colonoscopy surveillance on psychological well-being and health related quality of life (using the validated Psychological Consequences Questionnaire [23]). Third, factors individuals believed would lower the burden of colonoscopy surveillance. Individuals were asked to select 3 out of 18 statements which had previously been identified as main contributors to a more satisfactory colonoscopy [13]. Fourth, whether individuals considered quitting or postponing colonoscopy surveillance, and the reason(s) for this. Lastly, patients’ preferences regarding alternative (less-invasive) surveillance modalities, under the assumption that these would be non-inferior to colonoscopy in terms of accuracy in future.

### Compliance with surveillance

We investigated whether the patient-reported burden of colonoscopy surveillance was, amongst other factors, associated with compliance. Compliance data, including the reason(s) for non-compliance, were collected from the patients’ medical files. In line with a recent study [11], we considered an individual to be compliant with surveillance if the interval between the three most recent colonoscopies, and between the last colonoscopy and survey completion, did not differ more than six months from the recommended surveillance interval. The Dutch national guideline for Lynch syndrome recommends an interval of 2 years, however, in some individuals the treating physician opts for a one year interval (for example in those with a recent CRC or in those with a family member having had interval CRC). If three colonoscopies were not yet performed, (non-)compliance was determined based on all colonoscopies that individual had undergone.

### Statistics

Data were presented as medians with interquartile ranges (IQR) or as numbers with percentages. Factors that were associated with the overall patient-reported burden of colonoscopy surveillance based on univariable multinominal logistic regression (p < 0.20) were included in a multivariable model to identify independent associated factors. For the sake of this analysis, ‘extremely’ and ‘considerable’ burdensome were grouped together as well as ‘slightly’ and ‘not at all’. Both patient characteristics and endoscopy aspects (such as surveillance round, type of sedation, presence of neoplasia) were assessed; multicollinearity was taken into account. Following the same approach, multivariable binary logistic regression analysis was performed to identify variables associated with non-compliance to colonoscopy surveillance. To avoid overfitting of the models, the number of variables included did not exceed the number of events (non-compliance analysis) or the number of individuals in the smallest category (burden analysis) divided by ten. Statistical analyses were performed using IBM SPSS Statistics version 28. A p-value < 0.05 was considered statistically significant.

## Results

### Patient characteristics

**Fig. 1:**
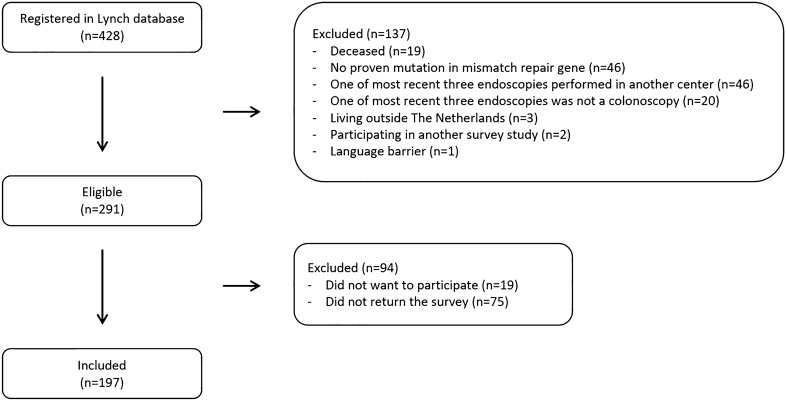
Flowchart showing the selection of the study population

Between November 2021 and March 2022, 197 of the 291 invited eligible individuals returned the survey (68% response rate, Fig. 1). The survey was rated as easy to perform in 86%, neither difficult nor easy in 13% and difficult in 1%. Of the respondents, 54% were female, 91% Caucasian and median age was 52 years (Table 1). Nine percent had a personal history of CRC and 27% had undergone bowel resection or gynaecologic surgery. The majority of individuals (60%) had undergone five or more colonoscopies in lifetime at the time of survey completion. The three most recent colonoscopies were performed under mild sedation (79%) with bowel preparation performed by Moviprep® (99%).

**Fig. 2:**
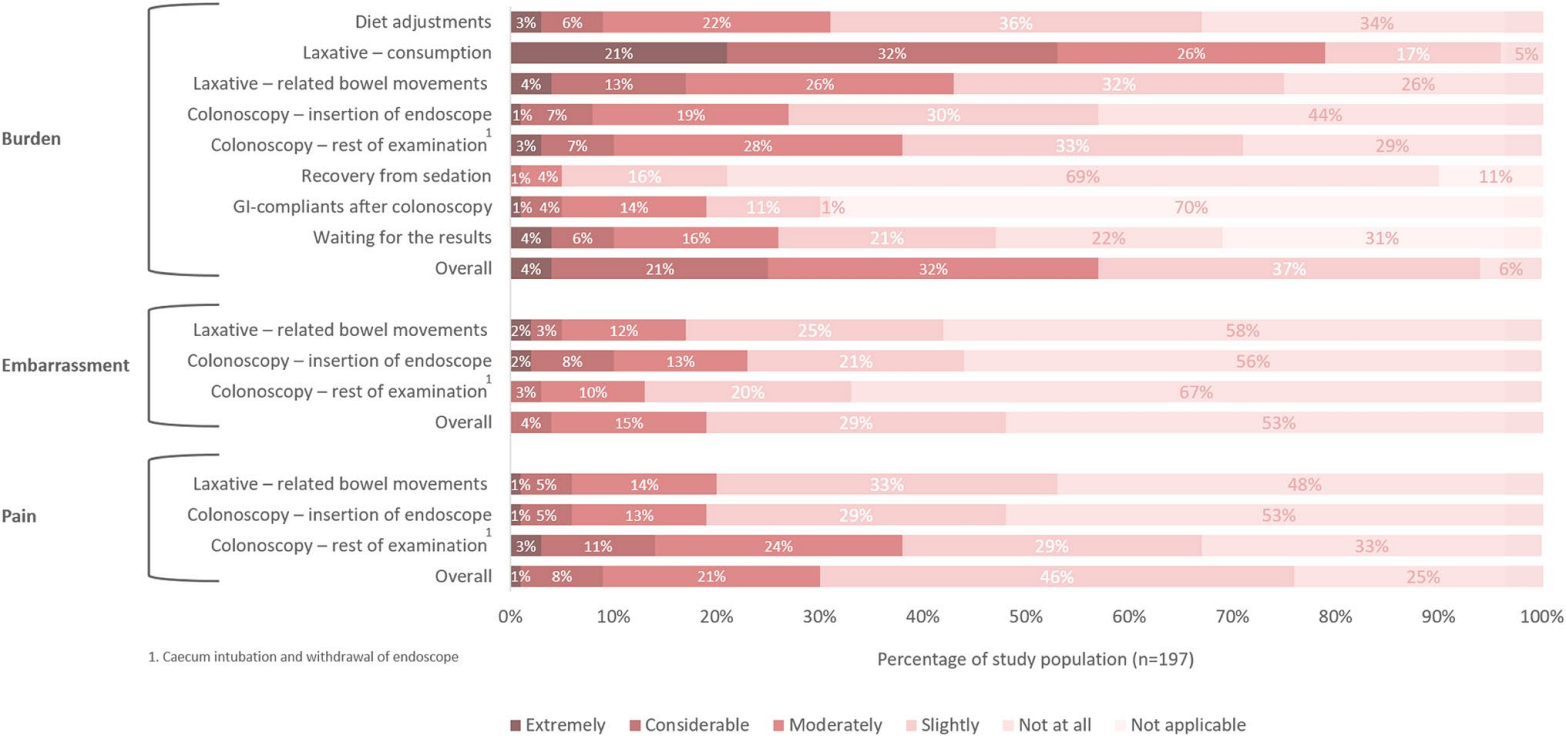
Impact of colonoscopy surveillance in Lynch syndrome

**Table 1:** Patient characteristics, n (%) or median (IQR)

Institute^a^	
Amsterdam UMC location AMC	131 (67%)
Amsterdam UMC location VUmc	65 (33%)
Age	52 (41–63)
< 40 years	45 (23%)
40–60 years	91 (46%)
> 60 years	61 (31%)
Female	106 (54%)
Caucasian	179 (91%)
Educational level^b^	
Low	16 (8%)
Medium	73 (37%)
High	107 (54%)
Employment status^b^	
Working full-time	88 (45%)
Working part-time	50 (25%)
Not working	58 (29%)
Net income household^b^	
Less than € 2.500 per month	42 (21%)
€ 2.500 to € 5.000 per month	58 (29%)
€ 5.000 or more per month	50 (25%)
Marital status^b^	
In a relationship	152 (77%)
Single or widow(er)	44 (22%)
Having children^b^	144 (73%)
Mutated MMR gene^b^	
MLH1	26 (13%)
MSH2	57 (29%)
MSH6	66 (34%)
PMS2/EPCAM	47 (24%)
Positive family history of CRC^b^	183 (93%)
Personal history of CRC	18 (9%)
History of abdominal surgery	
No	143 (73%)
Proctectomy or sigmoidectomy	5 (3%)
Hemicolectomy left or right	15 (8%)
Gynaecologic surgery	34 (17%)
Number of colonoscopies undergone in lifetime	
1–2	39 (20%)
3–4	39 (20%)
5–10	83 (42%)
> 10	36 (18%)
Time between most recent colonoscopy and survey	
1–4 weeks	15 (8%)
1–6 months	41 (21%)
7–12 months	35 (18%)
> 1 years	106 (54%)
Laxative used for most recent three colonoscopies^c^	
Moviprep ®	194 (99%)
Picoprep ®	21 (11%)
Kleanprep ®	10 (5%)
Sedation during most recent three colonoscopies	
No	5 (3%)
Mild sedation	156 (79%)
Deep sedation	36 (18%)
Neoplasia found with most recent three colonoscopies	61 (31%)

^a^In one subject colonoscopy surveillance had been performed in both institutions during study-period

^b^Cumulative percentage is not 100% due to some missing values

^c^Cumulative percentage is not 100% as some subjects had used different laxatives for the last three colonoscopies

### Impact of colonoscopy surveillance

Figure 2 shows the perceived pain, embarrassment and burden of different aspects of colonoscopy surveillance in individuals with Lynch syndrome. Overall, colonoscopy surveillance was experienced as extremely burdensome in 8/197 (4%), considerable burdensome in 42/197 (21%), moderately burdensome in 63/197 (32%), slightly burdensome in 73/197 (37%) and not burdensome in 11/197 (6%). Overall, forty-two individuals (21%) declared that colonoscopy surveillance does impact their quality of life. Particularly the bowel preparation was perceived as burdensome: 155/197 (79%) and 84/197 (43%) rated consumption of the laxative and laxation-related bowel movements, respectively, as moderately to extremely burdensome. Additionally, the majority of respondents (67%) indicated the bowel preparation as the most burdensome aspect of surveillance, predominantly the volume and/or taste of the laxative. Of note, these individuals indicating bowel preparation as most burdensome did not more often have a suboptimal Boston Bowel Preparation Scale (≤ 2 per segment) instead of an optimal score (≥ 3 per segment, logistic regression, p-value 0.683). The colonoscopy itself appeared to be most burdensome in 19% of respondents, and was considered moderately to extremely burdensome in 39%. The diet adjustments were considered moderately to extremely burdensome in 31%.

Multivariable analysis on the association between overall burden score and patient characteristics and endoscopy aspects, showed that individuals below 40 years of age experienced colonoscopy surveillance more often as moderately burdensome than individuals above 60 years of age (OR 3.23, p-value 0.047, Table S1 and Table S2).

Regarding the domain pain: colonoscopy surveillance was experienced as moderately to extremely painful in roughly one-third of respondents, which could be mainly attributed to the colonoscopy itself (rather than to the insertion of the endoscope or laxation-related bowel moments). Perceived embarrassment was low.

When individuals were asked about the effect of colonoscopy surveillance on psychological well-being and health related quality of life, most frequently reported feelings in the week preceding the procedure were being under strain (25% of individuals), nervous or strung up (21%) and worried about the future (18%).

### Compliance to colonoscopy surveillance

In total, non-compliance with the physician-set interval recommendation (being 2-yearly in 99% of individuals) was observed in 56/197 (28%) individuals. The non-compliance rate per centre was 22% (location VUmc) and 31% (location AMC). Non-compliance could mainly be attributed to the patient as the patient did not contact the hospital in time for a colonoscopy appointment (n = 35, 63%) or even postponed colonoscopy (n = 7, 13%). Other reasons for delay were pregnancy (n = 5, 9%) and hospital-related causes including the COVID-19 pandemic (n = 9, 16%). A multivariable model investigated whether certain factors were associated with patient-related non-compliance (n = 42). No association was found between non-compliance and colonoscopy surveillance being perceived as burdensome or as impacting quality of life (Table S3). However, we found that individuals with low and medium educational level as well as those having undergone ≤ 4 colonoscopies in lifetime were at higher risk for patient-related non-compliance (OR 7.41, OR 2.82, OR 20.68 – 34.77, respectively, Table S3). Additionally, being under surveillance at Amsterdam UMC location AMC, rather than location VUmc, was independently associated with non-compliance (OR 3.49).

When the 197 study participants were asked if they ever considered quitting or postponing colonoscopy surveillance, 41 (21%) answered positively. They reported that this was mainly because colonoscopy surveillance was burdensome (17/41), did not fit in their schedule (8/41) or reminded them of having Lynch syndrome (6/41). Of these 41 individuals, 20 were found to actually have patient-related surveillance delay. On the other hand, 22/42 individuals with patient-related delay indicated in the survey that they never considered quitting or postponing surveillance.

### Improvement of colorectal surveillance

Out of the 18 statements believed to lower the burden of colonoscopy [13], our cohort prioritised more acceptable bowel preparation regimens, shorter waiting times on the day of the colonoscopy, and a more personal and respectful approach of endoscopic staff (Table S4).

Additionally, the majority of individuals (61%) favoured alternative, less-invasive surveillance modalities: 34% of respondents favoured biomarkers, 19% imaging techniques (e.g. CT-scan or MRI-scan) and 8% capsule endoscopy; only 15% favoured a colonoscopy straightaway. Individuals who preferred alternative surveillance modalities experienced colonoscopy more often as moderately or considerable/extremely burdensome (OR 1.53 and 2.12, respectively, p-value < 0.001).

## Discussion

This study evaluated the impact of colonoscopy surveillance in 197 individuals with Lynch syndrome, a population with increased CRC risk requiring strict lifelong colonoscopy surveillance. We showed that surveillance was perceived as impacting quality of life in 21% of individuals and as moderately to extremely burdensome in 57%, particularly in those below age 40. Specifically burdensome were the volume and taste of the laxative and the laxation-related bowel movements. Other studies also indicated bowel preparation as the most burdensome aspect, independent of colonoscopy indication [9, 13, 15, 16]. Regarding the colonoscopy itself, we showed that perceived embarrassment was low whereas perceived pain modest, which is in line with previous research [14–16, 26]. Over 80% of our cohort did yet receive (adequate) sedation, hence, to reduce the pain scores the procedure itself needs to be further optimised. Lastly, we further explored surveillance behaviour in Lynch syndrome, including up-to-date non-compliance rates. We found that 28% of individuals had delayed colonoscopy surveillance and that an additional 10% considered quitting or postponing surveillance. Based on our results, we believe an effort should be made to improve acceptance of and compliance with colonoscopy surveillance in individuals with Lynch syndrome.

To potentially lower the burden of colonoscopy surveillance, participants prioritised more acceptable bowel preparation, shorter waiting times on the day of the colonoscopy, and a more personal and respectful approach of endoscopic staff. The vast majority of our cohort received bowel preparation by ascorbic acid-enriched polyethylene glycol (Moviprep®) as this was standard care in our centre, however, it has been shown that in both surveillance and other populations some might better tolerate the smaller volume preparations Picoprep® (magnesium citrate plus picosulphate) and Pleinvue® (polyethylene with higher ascorbate concentration), with non-inferior bowel cleansing quality and adenoma detection rates [27]. Hence, substituting Moviprep® by Picoprep® or Pleinvue® may be considered in clinical practice. Other studies also underlined the importance of a respectful and personal approach during the procedure, irrespective of its indication [13–15, 28, 29]. Familiarity with the endoscopist on the other hand seems to be particularly of value in populations requiring regular (surveillance) colonoscopies [13, 29].

A substantial proportion of our cohort (28%) showed non-compliance with 2-yearly colonoscopy surveillance, which was slightly higher than in other studies extracting “objective” compliance behaviour from medical files [8, 9, 11]. However, these studies did not take multiple surveillance rounds into account, used a less stringent definition for non-compliance or included patients with 2–3 yearly interval recommendations [8, 9, 11]. In our study, 76% of surveillance delays were patient-related. On multivariable analysis, patient-related surveillance delay was not associated with surveillance being experienced as burdensome, even though this was the main reason to consider quitting or postponing surveillance. Of the individuals considering quitting/postponing surveillance, only 50% actually had a patient-related delay. These findings might imply that individuals with Lynch syndrome regard colonoscopy as a life necessity and something that has to be endured, as has also been observed in patients with inflammatory bowel disease under strict surveillance [29]. Further, surveillance behaviour in Lynch syndrome, amongst other populations, can be explained and predicted using the Health Belief Model [30]. According to this model, surveillance behaviour is influenced by a patient’s 1) perceived susceptibility to the disease, 2) perceived severity of the disease, 3) perceived benefits of surveillance, 4) perceived barriers to action, 5) exposure to cues to action and 6) confidence in capability to succeed. Thorough exploration of these themes was beyond the scope of our study, though would be informative, hence qualitative research in this field is warranted.

In our study we gained insight into independent factors that prompt or deter individuals with Lynch syndrome to adhere to colonoscopy surveillance. First, patient-related delay was correlated with a history of ≤ 4 colonoscopies (OR 20.68 – 34.77). This might indicate that the first experiences with surveillance may determine whether or not individuals will participate in successive surveillance rounds, making it important that clinicians strive for a satisfactory first experience and that they provide clear information and interval recommendations. Secondly, non-compliance was associated with receiving surveillance at location AMC rather than location VUmc (OR 3.49). A major difference between both locations was that VUmc did have an automated recall system whereas AMC did not (see method section). We also found that of the patients having a patient-related delay, 50% indicated they never considered delaying surveillance. These findings suggest that individuals might not be aware of the recommended surveillance interval or of their surveillance being delayed, and that proactive management by a hospital recall system – for example coordinated by a centralised cancer registry such as the Netherlands Foundation for Detection of Hereditary Tumours – seem to facilitate better compliance. According to the previously mentioned Health Belief Model [30], such a reminder can serve as a “cue to action”. This also holds true for visiting a clinician who discusses the importance of surveillance, making it important that clinicians other than gastroenterologists (e.g. gynaecologists or general practitioners) recommend colonoscopy surveillance during consultation with someone having Lynch syndrome. The third identified predictor of non-compliance concerned low and medium educational level (OR 7.41 and OR 2.82, respectively), which is consistent with other studies on CRC screening [31–33]. Further research should explore why this sub-set of individuals shows lower compliance rates, and which strategies could enhance their compliance.

Another approach that could potentially improve compliance with colorectal surveillance in Lynch syndrome, as well as quality of life, is a less-invasive screening method that guides the need and/or timing of colonoscopy. Besides reducing the frequency of invasive and burdensome colonoscopic examination and bowel preparation, such a test would potentially reduce the interval CRC rate by selecting those requiring a colonoscopy at a shorter time interval. Our recent systematic review on non-endoscopic colorectal surveillance in Lynch syndrome concluded that non-invasive biomarkers may hold potential [34]. In the current study, participants expressed a clear preference for alternative less-invasive surveillance modalities, of which biomarkers were particularly favoured. Based on these observations, we believe (pre-)clinical studies in this field should be conducted.

To the best of our knowledge, this is the first study to date providing a comprehensive assessment of the impact of colonoscopy surveillance for Lynch syndrome in a large cohort of 197 individuals. Other strengths of our study lie in having used validated surveys which were formally re-validated in our population of interest, the high response rate of 68%, and 98% rating the survey as average or easy to perform. Moreover, non-compliance was not self-reported but extracted from medical files, and was determined based on three most recent colonoscopies; as the risk of CRC in Lynch syndrome is lifelong, CRC prevention is only effective if individuals adhere to multiple surveillance rounds over time. Only a small proportion of our cohort (20%) had undergone just one or two colonoscopies; these individuals were not excluded as this might have introduced selection bias, and we valued to also investigate surveillance burden and compliance in those just having started surveillance.

Several limitations should be acknowledged as well. First, our data were derived from a sample of mainly Caucasian individuals under surveillance in a single academic hospital, hence, may not be generalisable to other Lynch syndrome populations. Second, we did not gain insight into (factors predicting) uptake of the first colonoscopy following Lynch syndrome diagnosis. The third limitation concerns the possibility of selection bias, as non-compliers to surveillance are likely overrepresented among those who decline to participate in survey-based research [35]. This might imply that non-compliance rates were actually even greater than 28% in our practice.

In conclusion, our large study highlights that colonoscopy surveillance in Lynch syndrome is often experienced as burdensome, particularly in individuals below age 40. Moreover, we showed that colonoscopy surveillance is frequently delayed, and that the vast majority of the delays were for patient-related reasons. An effort should be made to improve acceptance of and compliance with colonoscopy surveillance in this population; potential interventions requiring further evaluation include a personalised bowel preparation regimen, personalised approach of endoscopic staff, short waiting times, clear information provision and interval recommendations, a hospital recall system and non-invasive biomarkers.

## Supplementary Information

Below is the link to the electronic supplementary material.

Supplementary file1 (DOCX 36 kb)

## Data Availability

The data that support the findings of this study are available from the corresponding author upon reasonable request.
