# Supplementary material for: Colonoscopy surveillance in Lynch syndrome is burdensome and frequently delayed
Source: Fam Cancer. 2023 May 12;22(4):403–11. doi: 10.1007/s10689-023-00333-4 (PMC10176312; doi:10.1007/s10689-023-00333-4)
Supplement: Supplementary file 1 — Supplementary file1 (DOCX 36 kb) [file 10689_2023_333_MOESM1_ESM.docx]

SUPPLEMENTARY FILE: Colonoscopy surveillance in Lynch syndrome is burdensome and frequently delayed.

**JOURNAL:** Familial Cancer

**AUTHORS**

Elsa L.S.A. van Liere, MD^1,2,3, 4^; Imke L. Jacobs, MD^1,4^; Evelien Dekker, MD PhD^2^; Maarten A.J.M. Jacobs, MD PhD^1^; Nanne K.H. de Boer, MD PhD^1,3,4^, Dewkoemar Ramsoekh, MD PhD^1,3,4^

**AFFILLIATIONS**

Department of Gastroenterology and Hepatology, Amsterdam University Medical Centers, Vrije Universiteit, De Boelelaan 1117, Amsterdam, The Netherlands.

^2^ Department of Gastroenterology and Hepatology, Amsterdam University Medical Centers, Meibergdreef 9, Amsterdam, The Netherlands.

^3^ Amsterdam Gastroenterology Endocrinology Metabolism (AGEM) Research Institute, Amsterdam, The Netherlands.

^4^ Vrije Universiteit, School of Medicine, Amsterdam, The Netherlands

**CORRESPONDING AUTHOR**

Elsa L.S.A. van Liere

e-mail: elsa.vanliere@amsterdamumc.nl

**Table S1.** Univariate analysis of variables associated with the overall burden of colonoscopy surveillance in Lynch syndrome (reference group: slightly/not at all burdensome)

| **Variable** | **Moderately burdensome**  Odds Ratio (95% - CI) p-value | **Extremely/considerable burdensome**  Odds Ratio (95% - CI) p-value |
| --- | --- | --- |
| **Institute**^1^  Amsterdam UMC location AMC  Amsterdam UMC location VUmc | 1.53 (0.75 – 3.13) 0.242  *Reference* | 1.01 (0.49 – 2.09) 0.987  *Reference* |
| **Age**^2^  < 40 years  40 – 60 years  > 60 years | 1.97 (0.78 – 5.00) 0.151  0.84 (0.39 – 1.80) 0.653  *Reference* | 1.94 (0.73 – 5.19) 0.187  0.86 (0.38 – 1.95) 0.716  *Reference* |
| **Gender**  Male  Female | 1.61 (0.84 – 3.12) 0.154  *Reference* | 0.62 (0.30 – 1.29) 0.202  *Reference* |
| **Educational level**  Low  Medium  High | 1.14 (0.31 – 4.26) 0.847  0.63 (0.31 – 1.26) 0.192  *Reference* | 1.64 (0.46 – 5.88) 0.448  0.50 (0.23 – 1.09) 0.082  *Reference* |
| **Employment status**  Working full-time  Working part-time  Not working | 2.03 (0.92 – 4.47) 0.080  0.79 (0.31 – 2.05) 0.630  *Reference* | 1.06 (0.46 – 2.43) 0.900  0.95 (0.38 – 2.33) 0.902  *Reference* |
| **Net income household**  Less than € 2.500 per month  € 2.500 to € 5.000 per month  € 5.000 or more per month | 0.55 (0.21 – 1.46) 0.233  0.96 (0.39 – 2.34) 0.920  *Reference* | 0.70 (0.25 – 1.95) 0.491  1.25 (0.49 – 3.23) 0.640  *Reference* |
| **Marital status**  Single or widow(er)  In a relationship | 1.60 (0.72 – 3.55) 0.248  *Reference* | 1.62 (0.70 – 3.76) 0.264  *Reference* |
| **Children**  Yes  No | 0.62 (0.29 – 1.31) 0.208  *Reference* | 0.64 (0.29 – 1.41) 0.267  *Reference* |
| **Mutated MMR gen**  MLH1  MSH2  MSH6  PMS2/ EPCAM | 1.13 (0.37 – 3.43) 0.836  0.87 (0.36 – 2.11) 0.750  0.93 (0.39 – 2.22) 0.863  *Reference* | 1.27 (0.38 – 4.29) 0.697  0.91 (0.34 – 2.45) 0.851  1.28 (0.50 – 3.28) 0.608  *Reference* |
| **Personal history of colorectal cancer**  No  Yes | 1.22 (0.38 – 3.93) 0.738  *Reference* | 0.95 (0.29 – 3.07) 0.928  *Reference* |
| **History of abdominal surgery**  No  Yes | 1.43 (0.68 – 3.01) 0.340  *Reference* | 1.28 (0.58 – 2.79) 0.542  *Reference* |
| **Subject’s perceived risk of developing colorectal cancer**^3^  Small  Intermediate  High | 0.63 (0.25 – 1.63) 0.343  0.61 (0.29 – 1.28) 0.194  *Reference* | 0.99 (0.37 – 2.63) 0.986  0.96 (0.44 – 2.09) 0.917  *Reference* |
| **Number of colonoscopies performed**  1 – 2  3 – 4  5 – 10  > 10 | 1.16 (0.40 – 3.31) 0.788  0.95 (0.33 – 2.72) 0.920  1.58 (0.64 – 3.92) 0.322  *Reference* | 1.51 (0.47 – 4.88) 0.489  1.22 (0.37 – 3.96) 0.743  2.06 (0.74 – 5.73) 0.168  *Reference* |
| **Time between most recent colonoscopy and survey**  1 – 4 weeks  1 – 6 months  7 – 12 months  > 1 years | 0.80 (0.22 – 2.94) 0.733  1.16 (0.51 – 2.63) 0.720  1.18 (0.47 – 2.96) 0.725  *Reference* | 0.97 (0.26 – 3.63) 0.968  0.76 (0.29 – 1.98) 0.570  1.44 (0.57 – 3.67) 0.442  *Reference* |
| **Sedation during most recent three colonoscopies**  No  Mild sedation  Deep sedation | 0.28 (0.03 – 3.11) 0.302  0.59 (0.25 – 1.43) 0.245  *Reference* | 0.31 (0.03 – 3.39) 0.334  0.49 (0.20 – 1.20) 0.119  *Reference* |
| **Neoplasia found with most recent three colonoscopies**  No  Yes | 1.43 (0.70 – 2.92) 0.330  *Reference* | 1.23 (0.58 – 2.61) 0.590  *Reference* |

1. Excluded from this analysis was the single subject in which colonoscopy surveillance had been performed in both institutions during study-period.

2. These age-groups were chosen based on the median age and corresponding interquartile range of our cohort, so each age-group would contain a sufficient number of subjects for a reliable statistical analysis.

3. Subject’s perceived risk of developing colorectal cancer was “small” in 33/197, “intermediate” in 67/197 and “high” in 97/197.

**Table S2.** Multivariate analysis of variables associated with the overall burden of colonoscopy surveillance in Lynch syndrome (reference group: slightly/not at all burdensome)

| **Variable^1^** | **Moderately burdensome**  Odds Ratio (95% - CI) p-value | **Extremely/considerable burdensome**  Odds Ratio (95% - CI) p-value |
| --- | --- | --- |
| **Age**^2^  < 40 years  40 – 60 years  > 60 years | 3.23 (1.02 – 10.25) 0.047  1.02 (0.44 – 2.36) 0.961  *Reference* | 2.90 (0.87 – 9.71) 0.083  1.10 (0.45 – 2.69) 0.833  *Reference* |
| **Gender**  Male  Female | 1.74 (0.86 – 3.52) 0.125  *Reference* | 0.63 (0.29 – 1.37) 0.247  *Reference* |
| **Educational level**  Low  Medium  High | 1.39 (0.34 – 5.68) 0.643  0.69 (0.34 – 1.44) 0.325  *Reference* | 2.00 (0.52 – 7.71) 0.316  0.49 (0.22 – 1.11) 0.086  *Reference* |
| **Number of colonoscopies performed**  1 – 2  3 – 4  5 – 10  > 10 | 0.65 (0.18 – 2.27) 0.496  0.71 (0.23 – 2.24) 0.559  1.42 (0.56 – 3.60) 0.465  *Reference* | 0.88 (0.23 – 3.40) 0.847  0.97 (0.28 – 3.44) 0.968  1.74 (0.60 – 5.02) 0.304  *Reference* |
| **Sedation during most recent three colonoscopies**  No  Mild sedation  Deep sedation | 0.28 (0.02 – 3.24) 0.307  0.68 (0.26 – 1.79) 0.431  *Reference* | 0.30 (0.03 – 3.46) 0.337  0.59 (0.22 – 1.59) 0.297  *Reference* |

1. To prevent overfitting of the model, only five variables could be included in the multivariate analysis. To select these five variables (out of the eight factors identified with univariate analysis), multivariate analysis was first performed with the variables most likely to influence experience of colonoscopy; e.g. age, gender, educational level and sedation. Next, the variables ‘employment status’, ‘perceived risk of CRC’ and ‘number of colonoscopies performed’ were added separately; ‘number of colonoscopies performed’ appeared to have the greatest influence on the model so was selected.

2. These age-groups were chosen based on the median age and corresponding interquartile range of our cohort, so each age-group would contain a sufficient number of subjects for a reliable statistical analysis.

**Table S3.** Multivariate analysis of variables associated with patient-related non-compliance to colonoscopy surveillance in Lynch syndrome

| **Variable** | **Univariate analysis**  Odds Ratio (95% - CI) p-value | **Multivariate analysis**  Odds Ratio (95% - CI) p-value |
| --- | --- | --- |
| **Institute**^1^  Amsterdam UMC location AMC  Amsterdam UMC location VUmc | 2.75 (1.14 – 6.66) 0.025  *Reference* | 3.49 (1.28 – 9.52) 0.015  *Reference* |
| **Age^2^**  < 40 years  40 – 60 years  > 60 years | 1.60 (0.57 – 4.48) 0.371  2.03 (0.87 – 4.77) 0.103  *Reference* | 0.90 (0.24 – 3.38) 0.877  1.61 (0.57 – 4.61) 0.372  *Reference* |
| **Gender**  Male  Female | 0.81 (0.40 – 1.61) 0.541  *Reference* | -  - |
| **Educational level**  Low  Medium  High | 4.67 (1.50 – 14.57) 0.008  2.68 (1.25 – 5.76) 0.011  *Reference* | 7.41 (1.66 – 33.12) 0.009  2.82 (1.22 – 6.53) 0.015  *Reference* |
| **Employment status**  Working full-time  Working part-time  Not working | 1.34 (0.57 – 3.15) 0.497  1.56 (0.60 – 4.06) 0.358  *Reference* | -  -  - |
| **Net income household**^3^  Less than € 2.500 per month  € 2.500 to € 5.000 per month  € 5.000 or more per month | 3.04 (1.08 – 8.59) 0.036  1.33 (0.47 – 3.76) 0.594  *Reference* | -  -  - |
| **Marital status**  In a relationship  Single or widow(er) | 0.74 (0.33 – 1.64) 0.453  *Reference* | -  - |
| **Children**  No  Yes | 0.92 (0.42 – 2.01) 0.832  *Reference* | -  - |
| **Mutated MMR gen**  MLH1  MSH2  MSH6  PMS2 / EPCAM | 0.98 (0.31 – 3.06) 0.967  0.56 (0.20 – 1.55) 0.266  1.22 (0.51 – 2.95) 0.656  *Reference* | -  -  -  - |
| **Personal history of colorectal cancer**  No  Yes | 1.43 (0.39 – 5.25) 0.587  *Reference* | -  - |
| **Subject’s perceived risk of developing colorectal cancer**  Small  Intermediate  High | 0.86 (0.31 – 2.40) 0.776  1.44 (0.68 – 3.07) 0.340  *Reference* | -  -  - |
| **Number of colonoscopies performed**  1 – 2  3 – 4  5 – 10  > 10 | 14.08 (1.70 – 116.47) 0.014  22.26 (2.75 – 180.00) 0.004  7.34 (0.92 – 58.40) 0.059  *Reference* | 20.68 (2.24 – 191.19) 0.008  34.77 (3.90 – 309.79) 0.001  7.19 (0.87 – 59.60) 0.068  *Reference* |
| **Sedation during most recent three colonoscopies**  No  Mild sedation  Deep sedation | 0.00 0.999  0.96 (0.40 – 2.32) 0.923  *Reference* | -  -  - |
| **Neoplasia found with most recent three colonoscopies**  No  Yes | 1.17 (0.55 – 2.50) 0.681  *Reference* | -  - |
| **Overall burden of colonoscopy surveillance**  Considerable to extremely burdensome  Moderately burdensome  Not at all to slightly burdensome | 1.21 (0.52 – 2.82) 0.655  0.83 (0.37 – 1.90) 0.664  *Reference* | -  -  - |
| **Impact of colonoscopy surveillance on quality of life**  Quality of life reduced  Quality of life not reduced | 1.05 (0.45 – 2.45) 0.904  *Reference* | -  - |

1. Excluded from this analysis was the single subject in which colonoscopy surveillance had been performed in both institutions during study-period.
2: These age-groups were chosen based on the median age and corresponding interquartile range of our cohort, so each age-group would contain a sufficient number of subjects for a reliable statistical analysis.

3. To prevent overfitting of the model, only four variables could be included in the multivariate analysis (out of the five factors identified with univariate analysis). In a sense income household is correlated with educational level (multicollinearity) so we decided to include either income household or educational level; chosen was educational level as this variable appeared to have the greatest influence in the univariate analysis.

**Table S4.** Factors individuals with Lynch syndrome believed would lower the burden of colonoscopy surveillance

| **Factor** | **Selected as main contributor to a more satisfactory colonoscopy^1^** |
| --- | --- |
| Easy access to doctor for substantial questions | 22 times selected |
| Clear follow-up appointments before leaving the hospital | 29 times selected |
| Being warned if painful part is to be expected | 40 times selected |
| Involvement in choice for sedation | 40 times selected |
| Easy access to treating doctor in case of emergency | 11 times selected |
| Good explanation of how to take the bowel preparation | 20 times selected |
| Good explanation of risks and complications | 22 times selected |
| Clear information leaflet | 27 times selected |
| Short waiting time on day of procedure | 57 times selected |
| Privacy during discussion of results | 21 times selected |
| Good explanation of reasons for colonoscopy | 11 times selected |
| Taste of the laxative | 115 times selected |
| Taking ample time for the colonoscopy | 33 times selected |
| Having one doctor that knows all about your situation | 38 times selected |
| Bond of trust between doctor and patient | 21 times selected |
| Respectful manner of the endoscopist | 55 times selected |
| Personal treatment during colonoscopy | 48 times selected |
| Good explanation of colonoscopy procedure | 20 times selected |

1. Individuals were asked to select 3 out of these 18 statements
